# Hepatitis B virus infection and associated risk factors among medical students in eastern Ethiopia

**DOI:** 10.1371/journal.pone.0247267

**Published:** 2021-02-19

**Authors:** Tewodros Tesfa, Behailu Hawulte, Abebe Tolera, Degu Abate

**Affiliations:** 1 Department of Medical Laboratory Sciences, College of Health and Medical Sciences, Haramaya University, Harar, Ethiopia; 2 Public Health and Policy Unit, School of Public Health, College of Health and Medical Sciences, Haramaya University, Harar, Ethiopia; 3 Epidemiology and Biostatistics Unit, School of Public Health, College of Health and Medical Sciences, Haramaya University, Harar, Ethiopia; Centre de Recherche en Cancerologie de Lyon, FRANCE

## Abstract

**Background:**

Hepatitis B virus (HBV) is a highly contagious pathogen that has become a severe public health problem and a major cause of morbidity and mortality, particularly in developing countries. Medical students are at high occupational risk during their training. However, no facility-based studies were found among medical students in eastern Ethiopia. Thus, this study aimed to investigate the seroprevalence of Hepatitis B Virus and associated factors among medical students in eastern Ethiopia.

**Methods:**

A facility-based cross-sectional study was conducted among 407 randomly selected medical students from March to June 2018. A pretested and structured questionnaire was used to collect data on socio-demographic characteristics and other risk factors. A 5ml blood was collected, and the serum was analyzed for Hepatitis B surface antigen (HBsAg) using the Instant Hepatitis B surface antigen kit. Data were entered using Epidata version 3.1 and analyzed using SPSS statistical packages version 22. Outcome and explanatory variables were described using descriptive summary measures. Binary and multivariable logistic regression was conducted at 95% CI and an association at P-value < 0.05 was declared statistically significant.

**Results:**

The seroprevalence of hepatitis B virus surface antigen was 11.5% (95%CI = 8.6, 14.7). Poor knowledge of universal precaution guideline (AOR = 2.58; 95% CI = [1.35–4.93]), history of needle stick injury (AOR = 2.11; 95% CI = [1.07–4.18]) and never been vaccinated for HBV (AOR = 2.34; 95% CI = [1.17–4.69]) were found statistically significantly associated with HBsAg positivity after multivariate analysis.

**Conclusion:**

Hepatitis B virus infection rate is high among health care trainees in eastern Ethiopia. Improvement at health care practice centers safety through training on universal precaution guidelines, and scaling up HBV vaccination is mandatory.

## Introduction

Hepatitis B virus (HBV) is a global public health concern particularly in sub-Saharan Africa, Western Pacific Region, and areas of Eastern Europe, with the prevalence ranging from 5–10%, and it is ranked the 15th cause of death in all-cause global mortality [1–4]. Approximately 15–25% of HBV infected people die due to liver cirrhosis, liver failure, or hepatocellular carcinoma [2]. As of 2014, around 2 billion people infected globally, and the carriage rate of chronic HBV varied from 0.1 and more than 20% [2].

Despite the effective implementation of vaccination programs, a large number of Asia and African countries are still classified as areas of high endemicity for HBV [5, 6]. The prevalence of HBV infection was >8% in parts of sub-Saharan Africa, such as West Africa [3]. Studies in Ethiopia indicate that all types of viral hepatitis origins are endemic, and the overall pooled prevalence of 6% HBV infection among the community [7]. Low vaccination coverage among adults higher than 18 years old (25.9%) in developing countries was indicated as the main risk factors for high endemicity [8].

Healthcare facilities are classified as hazardous and high-risk workplaces among several sectors because of the high incidence of work-related injuries and diseases [9, 10]. Health care workers ranging from direct care providers and practitioners to medical waste handlers are at risk of occupational hazard to infectious diseases like hepatitis B and C virus infection [11–14].

Medical trainees, especially in developing countries, have a very high risk of getting HBV infection during their training owing to the low HBV vaccine uptake rate and high rate of accidental exposure to body fluids [9, 15]. Reports in Africa indicate HBV infection rate among medical students reaches up to 31.5% [16]. A study in Northwest Ethiopia indicated up to 4.2% of HBV infection among medicine and health trainees [17].

Healthcare-associated vulnerability to infections due to inappropriate medical waste management practices and non-compliance with standard safety measures is a significant public health concern because it poses a high risk to workers’ health, family, and the community [9, 10, 18, 19]. For effective prevention and vaccination, adequate information is necessary for policymakers and program implementers. There are few studies conducted on HBV infection rates among high-risk groups such as medical students in Ethiopia. This study aimed to fill this information gap by conducting large-scale facility-based HBV prevalence study among medical students in eastern Ethiopia.

## Methods and materials

### Study design and setting

A facility-based cross-sectional study was conducted from March to June 2018 in medical training centers of Harari Region, Dire Dawa city administration, and west and east Hararghe zones of Oromia Regional State, eastern Ethiopia. According to the data obtained from the Respective health offices, there are 200 public health facilities (hospitals and health centers) in the study area. Eighteen hospitals (1 specialized hospital, 12 general hospitals, and six primary hospitals) serve as a practical site for medical students during the study period. Three higher educational institutions and several other private and regional owned training centers are conducting medical training in the eastern part of Ethiopia. The Eastern part of Ethiopia is known for its high population density, and it’s the route for the national import and export through Djibouti and Somali land ports.

### Participants and sampling procedures

The sample size of 422 students was determined, with an assumption of 80% power, 0.05% margin of error, and a design effect of 1.5. A stratified sampling technique was applied to select the study participants. Accordingly, hospitals were first stratified into three categories based on the national tier-structure (Primary, general, and specialized hospital). Then, by proportional allocation, a total of six hospitals (1 specialized hospital, three general hospitals, and two primary hospitals) were selected randomly by lottery. We have allocated study participants proportionally to the selected hospitals based on the number of students attaching. Participants were recruited by systemic random sampling methods using a list of students assigned to each hospital as a sampling frame. Hiwot Fana Specialized University Hospital (145 students), Karamara General Hospitals (55 students), Dilchora General Hospital (90 students), Chiro General Hospital (67 students), Sabian Primary Hospital (30 students), and Chelenko Primary Hospital (35 students) were the study sites. Students who have no exposure to patients/ biological substance were excluded from the study.

### Data collection and data quality control

Five Nurses and five Public health professionals facilitated data collection using a pretested and structured questionnaire. The questionnaire was developed after reviewing different literature [20–23], translated into the local language (Afan Oromo and Amharic), and pretested 5% of the sample size working at Jugol hospital. The variables included socio-demographic characteristics, smoking, nutrition, alcohol consumption, history of liver disease, vaccination to HBV, potential risk factors (exposure to blood or body fluid in the eye, nose, needle stick and sharp injury, training on infection prevention and the wearing of gloves). There was strict supervision during data collection and checked for completeness each day. Furthermore, we have checked for the expiry date and functionality of the material used for blood sample collection (alcohol, cotton, blood collection tube, and syringe). Kits verified with a known HBsAg positive sample from Hiwot Fana Specialized University Hospital for functionality.

#### Blood sample

5ml of venous blood sample was collected by a laboratory professional into tubes aseptically after obtaining written consent from study participants. The blood samples were labeled with unique identification numbers and clotted. Serum was separated by centrifugation at 3000 r/min for 5 min and placed into Eppendorf tubes. The serological tests were performed using hepatitis B surface antigen rapid test kits (Guangzhou Wondfo Biotech Co., Ltd (Wondfo)) at the study site (hospital). This test strip has a sensitivity of 96.2% and specificity of 99.3%.

### Measurements

#### HBsAg positive

Samples which were reactive/ positive for the test were considered as HBsAg positive.

#### Knowledge of universal precautions

Knowledge was assessed by questions focusing on universal precaution. Each response was scored as ‘yes’ or ‘no’. The scoring range of the questionnaire was 10 (largest) to 0 (smallest). A cut off level of <7 was considered as poor whereas > = 12 was considered as good knowledge about universal precaution.

#### Needle stick injury

The penetration of the skin by a hypodermic needle or other sharp object that has been in contact with blood, tissue or other body fluids before the exposure.

### Data processing and analysis

Data entered using Epidata version 3.1 and exported to SPSS version 22 statistical packages for cleaning and analysis. Data presented using descriptive and inferential statistics. Chi-square and Fisher exact tests were used to assess the association between outcome and independent variables. Bivariate and multivariate logistic regression analysis was employed at a 95% confidence interval to determine the presence of an association between explanatory variables and the seropositivity of HBsAg. P-value at < 0.05 at 95% CI was taken statistically significant and the Hosmer Lemeshow goodness of fit was used for model fitness.

### Ethical considerations

The Institutional Health Research Ethics Review Committee (IHRERC) of Haramaya University, College of Health and Medical Sciences (CHMS) has approved the study (IHRERC/093/2017). Respective institutions permitted, and participants were informed of the study purposes, procedures, potential risks, and benefits. Then, every participant gave s written and signed consent. Names and identity numbers of participants were not included in the questionnaires for the sake of confidentiality. Results were communicated to study subjects based on their request, and counseling was given for participants who were infected with the hepatitis B virus, and they were referred for further workup and treatment for hepatitis B virus infection.

## Result

A total of 407 (96.44%) medical students have participated in this study. Two hundred forty-three (59.7%) of these participants were males. The median age of the study participant was 24 years old (IQR = 5), and 65.5% of the respondents attend nursing education. Out of the total study participants, 47.7%) were Muslim and 48.1%) were Oromo ethnic groups (**Table 1**).

**Table 1:** Sociodemographic characteristics of the study participant, eastern Ethiopia, 2018.

Variables	Category	Frequency	Percent (%)
**Sex**	Male	243	59.7
	Female	164	40.3
**Marital Status**	Single/unmarried	313	76.9
	Married	85	20.9
	Divorced	9	2.2
**Year of Study**	Graduate class	126	31.0
	Third year class	216	53.1
	Second year class	54	13.3
	First year class	11	2.7
**Religion**	Muslim	194	47.7
	Orthodox	162	39.8
	Protestant	49	12
	Others	2	0.5
**Ethnicity**	Oromo	210	48.1
	Amhara	146	36.8
	Harari	17	7.1
	Tigray	15	4.3
	Others	19	3.7

### Health care trainees’ knowledge on universal precaution guidelines, training, and occupational exposure status

**Fig 1:**
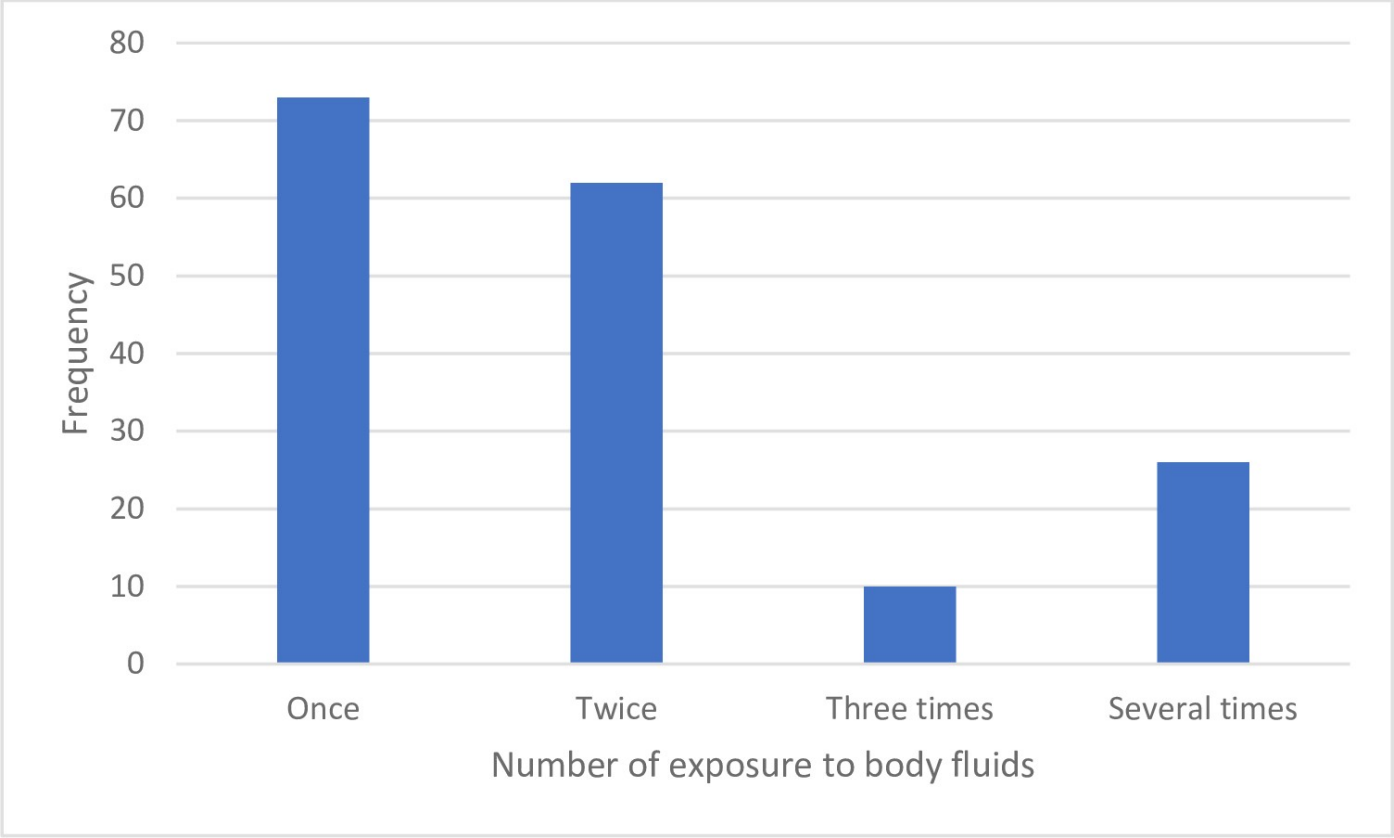
Frequency of exposure to body fluids among medical students, eastern Ethiopia, 2018.

In this study, 70.5% of the study participants have good knowledge of universal precaution guideline, and 42.0% have participated in any training program about infection prevention or universal precaution. The majority of students (62.7%) have participated in the collection of patients’ blood, and 88.5% of respondents said that they cleaned their hands after touching or collecting blood or other body fluids using plain water and soap or alcohol-based hand rubbing. Three hundred twenty-five (80%) participants said they did wear personal protective equipment during procedures. Two hundred forty-nine study participants (61.2%) wear gloves consistently, whereas one hundred fifty-eight (38.8%) wear gloves intermittently as personal protective equipment. On the other hand, about half (42.0%) of the study participants have a history of exposure to any body fluids, like waste contaminated by body fluids (blood, peritoneal, pericardial, pleural, synovial, CSF amniotic fluids, and others). Among those exposed to body fluids, 42.69% of them had at least one exposure (**Fig 1**), and 76.61% washed the splash of body fluids with alcohol and/or soap.

Among all study subjects, 48.6% said the needle should be recapped or bent after use. Two hundred twenty-five (55.3%) study subjects have frequently practiced needle recapping, and 33.9% had a history of needle stick injury. The majority of the respondents (57.2%) ever cared for hepatitis virus-positive patients, and 11.3% had participated in surgical operations. Around 15% of the trainees have received blood donation, and 4.4% have been diagnosed with jaundice and/or liver diseases.

Fifty-two (12.8%) and one hundred ten participants (27%) had a history of tattooing and tooth extraction, respectively. From the total participants, 3.4% had multiple sexual partners in the last year, and 4.4% had a family history of chronic liver diseases, and 48.4% have been vaccinated for HBV.

### Prevalence of hepatitis B infection among study subjects

According to this study, 11.5% (95%CI: 8.6–14.7) of the respondents found positive for HBsAg. Among subjects diagnosed with HBsAg, 76.20% are males, and it was not statistically different from females (P = 0.08), and 55.32% are third-year clinical practitioner students. The majority of positive cases have poor knowledge of universal precaution guidelines and have not participated in a training of universal precaution, 53.19%, and 74.47%, respectively. Also, 65.95% of positive cases are among those who do not commonly use gloves as personal protective equipment. Neither sociodemographic characteristics nor field of study have statistically significant differences between HBV negative and HBV positive medical students.

### Factors associated with HBV infection among health care students

Variable with P-value less than 0.2 in the bivariate analysis, knowledge about personal protective equipment (PPE), exposure to body fluids, and history of needle stick injury, vaccination status, history of tooth extraction, family history of chronic liver disease, and history of operation/surgery, were selected for multivariable analysis model.

In the multivariate binary logistic regression, after controlling for confounders, the odds of being positive for HBsAg infection is almost three times higher (AOR = 2.58, 95% CI: 1.35–4.93) among medical students who have poor knowledge of universal precaution guidelines (**Table 2**). Health care students who have a history of needle stick injury were almost two times (AOR = 2.11, 95% CI: 1.07–4.18) more likely to be positive for HBsAg than those who have no history. Similarly, medical students who have never vaccinated for HBV were two times (AOR = 2.34, 95% CI: 1.17–4.69) more likely to be positive for HBsAg (**Table 2**).

**Table 2:** Factors associated with HBsAg positive status among medical students at public health facilities in eastern Ethiopia, 2018 (n = 407).

Variables	Response	HBsAg status		COR [95%CI]	AOR [95%CI]	P-value
		Positive	Negative			
**Knowledge about UP guideline**	yes	25	262	1	1	
	no	22	98	2.35 (1.27 4.37)	2.58[1.35 4.93]	0.004
**Exposure to body fluids ungloved**	Yes	26	145	1.84(1.00 3.39)	1.49[0.73 3.04]	0.27
	No	21	215	1	1	
**History of needle stick injury**	Yes	23	115	2.20(1.19 4.05)	2.11[1.07 4.18]	0.03
	No	24	245	1	1	
**History of operation/surgery**	yes	2	44	0.46 (0.16 1.38)	0.29[0.06 1.33]	0.11
	No	45	316	1	1	
**History of tooth extraction**	Yes	17	93	1.63(0.86 3.09)	1.06[0.51 2.21]	0.90
	No	30	67	1	1	
**Family history of chronic liver diseases**	Yes			2.30(0.72 7.30)	3.15[0.88 11.33]	0.08
	No			1	1	
**Ever vaccinated for HBV**	Yes	15	182	1	1	
	No	32	178	2.18 (1.14 4.17)	2.34[1.17 4.69]	0.02

## Discussion

This study found that the seroprevalence of hepatitis B surface antigen among medical students was 11.5%. Knowledge of universal precaution, experiencing needle stick injury, vaccination for HBsAg were determinants of HBV infection among medical students.

The seroprevalence of hepatitis B surface antigen in the current study is lower compared to a study report from Nigeria (31.5%) [16], whereas it is higher than Saudi Arabia (0.41%) [24]. It is, however, relatively consistent with studies done among health professionals in Uganda (8.1%) [25], Tanzania (7.0%) [26], and southern Ethiopia (7.3%) [20]. The finding of this study also goes with the report of articles in which high prevalence (equal to or greater than eight 8%) of hepatitis B infection occurs in countries like North America, South America, Sub-Saharan Africa, and most Asian countries [27–29]. The high seroprevalence could be due to inadequate knowledge, attitude, and practice of infection prevention packages. In the current study setting, the involvement of a large number of facilities, lack of training on the universal precaution guidelines, poor medical waste management system may contribute to the occurrence of infection.

On the other hand, 48.4% of the study participants have received at least one dose of HBV vaccine. This finding is higher than a study done in Cameroon (11.4%) [30] but lower than Northern Tanzania (67.4%) [31], Kenya (80%) [32], Malaysia (95.91%) [33] and Pakistan (73.42%) [34]. This difference could be due to the unavailability of the HBV vaccine for adults above 18 years in Ethiopia [35]. However, it is similar to a study conducted in southwestern Niger (48.5%) [36].

Medical students with poor knowledge of universal precaution guidelines were about three times more likely to be infected by HBV than their counterparts. Previous studies in Nigerian universities indicated that medical students who were not aware of medical practice guidelines were more likely to be predisposed to HBV infection [37] due to occupational hazards like needlestick injuries [38]. Similarly, a study in Bhatia Medical and Dental College, Mirpur Khasas showed despite a high level of knowledge regarding Needlestick injury (NSI) and cross-infection, there is 63% NSI due to low awareness about prevention that mostly occurred during needle recapping mainly due to lack of clinical skills and experience [39]. Knowledge is essential for effecting changes in behavior, and the accessibility of this knowledge intends to improve the recognition of health-related behavior like practicing universal precaution guidelines for the prevention of infection. Thus, medical students should be well trained for infection prevention before employed in the practice/ attachment.

This study indicated a greater than 2-fold higher risk for medical students to acquire HBV infection if they ever had needle stick injury. This finding is in line with studies in northeast Ethiopia [17] and Nigeria [37] that showed medical students are at increased risk of HBV infection due to occupational needle stick injury. Accidental ‘needle prick’ and ‘cut from sharp’ while recapping needles or detaching needles were the most common type of exposure identified during medical care or practice [19]. Needle sticks and related injuries often get unnoticed [40, 41]. Previous studies showed HBV transmissions mainly resulted from non-compliance with aseptic techniques such as the use of inadequately sterilized needles and medical instruments and the reuse of disposable needles and syringes [42, 43].

Medical students with no vaccination for HBV were two times more likely to be positive for HBsAg. This finding is similar to a study in Northwest Ethiopia, which indicated medical trainees are at a very high risk of contracting HBV infection, owing to the low HBV vaccine uptake rate [15]. A study in Cameroon indicated a high incidence of accidental exposure to blood, and low HBV vaccination uptake among medical students led to a high occupational risk of HBV infection [44]. Several other studies also indicated low immunization status against the hepatitis B virus was associated with a higher chance of hepatitis B virus (HBV) infection [45]. Ethiopia has a universal vaccination program for HBV among children with other vaccination panels. When it comes to medical trainees, there is no standard guideline to enforce educational institutions to provide HBV vaccine in pre-service training even-though many institutions are trying to administer the vaccine to medical trainees.

### Strength and limitation of the study

This study has certain limitations, including insufficient immunoassay logistics supply, which limited this study to provide data on IgM anti-HBc and HBeAg markers. That affected the study in classifying whether infections are acute or chronic and whether the degree of infectivity correlates with a high level of HBV replication or not. Besides, this research cannot establish the temporal relation of whether the participants acquired the infection before hired in the health institutions or after. Despite these limitations, the representativeness can be appreciated as it has included large scale health facilities and geographic areas.

## Conclusions

This study revealed a high prevalence of hepatitis B virus infection among medical students, mainly due to poor knowledge about universal precaution guidelines, needle stick injury, and being unvaccinated. Institutions have to provide training on universal precaution guidelines and safety procedures effectively. We recommend large scale vaccination for health care students despite good vaccination coverage compared to previous studies. In general, on training education occupational and personal protection should be provided to health care students in this region of Ethiopia.

## Supporting information

S1 Data (XLS)

## References

[1] Ginzberg D, Wong RJ, Gish R. Global HBV burden: guesstimates and facts. *Hepatology International*. 2018;12(4):315–29. 10.1007/s12072-018-9884-8 30054801

[2] Lavanchy D, Kane M. Global Epidemiology of Hepatitis B Virus Infection In: Liaw Y-F, Zoulim F, editors. Hepatitis B Virus in Human Diseases. Cham: Springer International Publishing; 2016 p. 187–203.

[3] Stasi C, Silvestri C, Voller F. Emerging Trends in Epidemiology of Hepatitis B Virus Infection. *J Clin Transl Hepatol*. 2017;5(3):272–6. 10.14218/JCTH.2017.00010 28936408 PMC5606973

[4] WHO. Global Hepatitis Report 2017. Geneva: World Health Organization; 2017 Licence: CC BY-NC-SA 3.0 IGO. CIP data are available at http://apps.who.int/iris. ISBN 978-92-4-156545-5. 2017.

[5] McNaughton AL, Lourenço J, Bester PA, Mokaya J, Lumley SF, Forde D, et al HBV seroepidemiology data for Africa provides insights into transmission and prevention. *bioRxiv*. 2019:654061.

[6] Tamandjou CR, Maponga TG, Chotun N, Preiser W, Andersson MI. Is hepatitis B birth dose vaccine needed in Africa? *The Pan African medical journal*. 2017;27(Suppl 3):18–. 10.11604/pamj.supp.2017.27.3.11546 29296153 PMC5745936

[7] Belyhun Y, Maier M, Mulu A, Diro E, Liebert UG. Hepatitis viruses in Ethiopia: a systematic review and meta-analysis. *BMC Infect Dis*. 2016;16(1):761 10.1186/s12879-016-2090-1 27993129 PMC5168848

[8] Lu P-j, O’Halloran AC, Williams WW, Nelson NP. Hepatitis B vaccination coverage among adults aged ≥18 years traveling to a country of high or intermediate endemicity, United States, 2015. *Vaccine*. 2018. 10.1016/j.vaccine.2018.03.031 29716773

[9] Aluko OO, Adebayo AE, Adebisi TF, Ewegbemi MK, Abidoye AT, Popoola BF. Knowledge, attitudes and perceptions of occupational hazards and safety practices in Nigerian healthcare workers. *BMC research notes*. 2016;9(1):71 10.1186/s13104-016-1880-2 26852406 PMC4744628

[10] Burton JL. Health and safety at necropsy. *Journal of Clinical Pathology*. 2003;56(4):254 10.1136/jcp.56.4.254 12663635 PMC1769932

[11] Niu S. Ergonomics and occupational safety and health: An ILO perspective. *Applied Ergonomics*. 2010;41(6):744–53. 10.1016/j.apergo.2010.03.004 20347066

[12] Nkonge Njagi A, Mayabi Oloo A, Kithinji J, Magambo Kithinji J. Knowledge, Attitude and Practice of Health-Care Waste Management and Associated Health Risks in the Two Teaching and Referral Hospitals in Kenya. *Journal of Community Health*. 2012;37(6):1172–7. 10.1007/s10900-012-9580-x 22752531

[13] Schulte PA. Emerging Issues in Occupational Safety and Health. *International Journal of Occupational and Environmental Health*. 2006;12(3):273–7. 10.1179/oeh.2006.12.3.273 16967836

[14] Tziaferi SG, Sourtzi P, Kalokairinou A, Sgourou E, Koumoulas E, Velonakis E. Risk Assessment of Physical Hazards in Greek Hospitals Combining Staff’s Perception, Experts’ Evaluation and Objective Measurements. *Safety and Health at Work*. 2011;2(3):260–72. 10.5491/SHAW.2011.2.3.260 22953210 PMC3430906

[15] Abdela A, Woldu B, Haile K, Mathewos B, Deressa T. Assessment of knowledge, attitudes and practices toward prevention of hepatitis B virus infection among students of medicine and health sciences in Northwest Ethiopia. *BMC research notes*. 2016;9(1):410 10.1186/s13104-016-2216-y 27543117 PMC4992214

[16] Tula M, Iyoha O. A Cross-sectional Study on the Sero-prevalence of Hepatitis B Surface Antigen (HBsAg) among Apparently Healthy Students of a Tertiary Institution in North-Eastern Nigeria *Int*. *J*. *Trop*. *Dis*. *Health*. 2015;7(3):102–8.

[17] Demsiss W, Seid A, Fiseha T. Hepatitis B and C: Seroprevalence, knowledge, practice and associated factors among medicine and health science students in Northeast Ethiopia. *PLOS ONE*. 2018;13(5):e0196539 10.1371/journal.pone.0196539 29763447 PMC5953438

[18] Coker A, Sangodoyin A, Sridhar M, Booth C, Olomolaiye P, Hammond F. Medical waste management in Ibadan, Nigeria: Obstacles and prospects. *Waste Management*. 2009;29(2):804–11. 10.1016/j.wasman.2008.06.040 18835151

[19] Shivalli S, Sowmyashree H. Occupational exposure to infection: a study on healthcare waste handlers of a tertiary care hospital in South India *The Journal of the Association of Physicians of India*. 2015;63(11):24–7. 29900707

[20] Geberemicheal A, Gelaw A, Moges F, Dagnaw M. Seroprevalence of hepatitis B virus infections among health care workers at the Bulle Hora Woreda Governmental Health Institutions, Southern Oromia, Ethiopia. *Journal of Environmental and Occupational Health*. 2013;2(1):9–14.

[21] Pido B, Kagimu M. Prevalence of hepatitis B virus (HBV) infection among Makerere University medical students. *African health sciences*. 2005;5(2):93–8. 16006214 PMC1831914

[22] Mesfin YM, Kibret KT. Assessment of knowledge and practice towards hepatitis B among medical and health science students in Haramaya University, Ethiopia. *PloS one*. 2013;8(11):e79642 10.1371/journal.pone.0079642 24278151 PMC3836877

[23] Singh A, Jain S. Prevention of hepatitis B; knowledge and practices among medical students. *Healthline*. 2011;2(2):8–11.

[24] Medani KE, Al Fehaid F, Abdalla SM, Bashir AA, Al Mansour M, Yousif E. The prevalence of hepatitis B among medical students, Majmaah University, Kingdom of Saudi Arabia. *Int J Pharm Med Res*. 2015;3:191–4.

[25] Ziraba AK, Bwogi J, Namale A, Wainaina CW, Mayanja-Kizza H. Sero-prevalence and risk factors for hepatitis B virus infection among health care workers in a tertiary hospital in Uganda. *BMC Infect Dis*. 2010;10(1):191. 20587047 10.1186/1471-2334-10-191 PMC2910699

[26] Mueller A, Stoetter L, Kalluvya S, Stich A, Majinge C, Weissbrich B, et al Prevalence of hepatitis B virus infection among health care workers in a tertiary hospital in Tanzania. *BMC Infect Dis*. 2015;15(1):386. 26399765 10.1186/s12879-015-1129-z PMC4581415

[27] MacLachlan JH, Cowie BC. Hepatitis B virus epidemiology. *Cold Spring Harb Perspect Med*. 2015;5(5):a021410–a. 10.1101/cshperspect.a021410 25934461 PMC4448582

[28] Schweitzer A, Horn J, Mikolajczyk RT, Krause G, Ott JJ. Estimations of worldwide prevalence of chronic hepatitis B virus infection: a systematic review of data published between 1965 and 2013. *The Lancet*. 2015;386(10003):1546–55. 10.1016/S0140-6736(15)61412-X 26231459

[29] Zampino R, Boemio A, Sagnelli C, Alessio L, Adinolfi LE, Sagnelli E, et al Hepatitis B virus burden in developing countries. *World J Gastroenterol*. 2015;21(42):11941–53. 10.3748/wjg.v21.i42.11941 26576083 PMC4641116

[30] Bilounga Ndongo C, Eteki L, Siedner M, Mbaye R, Chen J, Ntone R, et al Prevalence and vaccination coverage of Hepatitis B among healthcare workers in Cameroon: A national seroprevalence survey. *Journal of Viral Hepatitis*. 2018;25(12):1582–7. 10.1111/jvh.12974 30047565 PMC6717319

[31] Shao ER, Mboya IB, Gunda DW, Ruhangisa FG, Temu EM, Nkwama ML, et al Seroprevalence of hepatitis B virus infection and associated factors among healthcare workers in northern Tanzania. *BMC Infect Dis*. 2018;18(1):474 10.1186/s12879-018-3376-2 30241503 PMC6151054

[32] Kisangau EN, Awour A, Juma B, Odhiambo D, Muasya T, Kiio SN, et al Prevalence of hepatitis B virus infection and uptake of hepatitis B vaccine among healthcare workers, Makueni County, Kenya 2017. *Journal of Public Health*. 2018. 10.1093/pubmed/fdy186 30351408

[33] Shin LP, Appasamy ES, Sandhu RS, Appalanaidu TL, Samaratunga DJW. A Cross Sectional Study on Knowledge, Attitude and Practice of Hepatitis B Vaccination Among Medical Students in Malaysia. *Open Science Journal of Clinical Medicine*. 2019;7(3):83.

[34] Attaullah S, Khan S, Naseemullah, Ayaz S, Khan SN, Ali I, et al Prevalence of HBV and HBV vaccination coverage in health care workers of tertiary hospitals of Peshawar, Pakistan. *Virology Journal*. 2011;8(1):275 10.1186/1743-422X-8-275 21645287 PMC3121707

[35] Anagaw B, Shiferaw Y, Anagaw B, Belyhun Y, Erku W, Biadgelegn F, et al Seroprevalence of hepatitis B and C viruses among medical waste handlers at Gondar town Health institutions, Northwest Ethiopia. *BMC research notes*. 2012;5(1):55. 22264306 10.1186/1756-0500-5-55 PMC3274440

[36] Abiola A-HO, Agunbiade AB, Badmos KB, Lesi AO, Lawal AO, Alli QO. Prevalence of HBsAg, knowledge, and vaccination practice against viral hepatitis B infection among doctors and nurses in a secondary health care facility in Lagos state, South-western Nigeria. *Pan African Medical Journal*. 2016;23(1). 10.11604/pamj.2016.23.160.8710 PMC4894726 27303576

[37] Okeke E, Ladep N, Agaba E, Malu AJNJM. Hepatitis B vaccination status and needle stick injuries among medical students in a Nigerian university. 2008;17(3):330–2. 10.4314/njm.v17i3.37404 18788262

[38] Solomon O, Mahafroz K, Mashor M, Arome F, Neha DJIjos, publications r. Prevalence of HBV and HIV among students and staffs at the University of Jos, Nigeria: Results from a medical outreach screening program. 2014;4(11):2250–3153.

[39] Ali I, Hameed F, Maqbool A, Kazim M, Aslam MA, Siddiqui SU, et al Incidence of Needle Stick Injury (NSI) among Dental Students and Dental House Officers of Bhitai Medical and Dental College, Mirpur Khas. 2019;5(1).

[40] Ream PSF, Tipple AFV, Salgado TA, Souza ACS, Souza SMB, Galdino-Júnior H, et al Hospital housekeepers: Victims of ineffective hospital waste management. 2016;71(5):273–80. 10.1080/19338244.2015.1089827 26359679

[41] Thakur A, Toppo M, Pal. Occupational Exposure to Needlestick and Sharp Injuries among Hospital Waste Handlers in Selected Government Health Facilities of Bhopal District *Int J Health Sci Res*. 2015;5(5):37–43.

[42] Hauri AM, Armstrong GL, Hutin YJJIjoS, AIDS. The global burden of disease attributable to contaminated injections given in health care settings. 2004;15(1):7–16. 10.1258/095646204322637182 14769164

[43] Makuza JD, Rwema JOT, Ntihabose CK, Dushimiyimana D, Umutesi J, Nisingizwe MP, et al Prevalence of hepatitis B surface antigen (HBsAg) positivity and its associated factors in Rwanda. *BMC Infect Dis*. 2019;19(1):381 10.1186/s12879-019-4013-4 31053097 PMC6499977

[44] Noubiap JJN, Nansseu JRN, Kengne KK, Tchokfe Ndoula S, Agyingi LA. Occupational exposure to blood, hepatitis B vaccine knowledge and uptake among medical students in Cameroon. *BMC Medical Education*. 2013;13(1):148 10.1186/1472-6920-13-148 24200149 PMC3874660

[45] Otovwe A, Adidatimi PO. Knowledge, Attitude and Practice of standard precaution among Health Care Workers in Federal Medical Centre Yenagoa, Nigeria, *IOSR Journal of Pharmacy and Biological Sciences (IOSR-JPBS)*e-ISSN:2278-3008, p-ISSN:2319-7676. Volume 12, Issue 4 Ver. III, PP 79–86, www.iosrjournals.org 2017 10.4314/ejhs.v27i4.5 29217936 PMC5615023

